# Conjunctive hierarchical secret sharing by finite geometry

**DOI:** 10.1007/s10623-024-01496-6

**Published:** 2024-10-16

**Authors:** Máté Gyarmati, Péter Ligeti, Peter Sziklai, Marcella Takáts

**Affiliations:** 1 Department of Computeralgebra, Eötvös Loránd University, Budapest, Hungary; 2 HUN-REN Alfréd Rényi Institute of Mathematics, Budapest, Hungary; 3 HUN-REN-ELTE GAC Research Group, Budapest, Hungary; 4 Department of Computer Science, Eötvös Loránd University, Budapest, Hungary

**Keywords:** Secret sharing, Threshold schemes, Finite geometry, 51E99, 94A60

## Abstract

Secret sharing is a general method for distributing sensitive data among the participants of a system such that only a collection of predefined qualified coalitions can recover the secret data. One of the most widely used special cases is threshold secret sharing, where every subset of participants of size above a given number is qualified. In this short note, we propose a general construction for a generalized threshold scheme, called conjunctive hierarchical secret sharing, where the participants are divided into disjoint levels of hierarchy, and there are different thresholds for all levels, all of which must be satisfied by qualified sets. The construction is the first method for arbitrary parameters based on finite geometry arguments and yields an improvement in the size of the underlying finite field in contrast with the existing results using polynomials.

## Introduction

*Secret sharing* was first introduced by Shamir [7] and Blakley [2] to distribute sensitive information, called the *secret*, between the participants $\mathcal {P}$ of the protocol in a way that only some predefined coalitions of the participants, called *qualified subsets* can recover the secret. The distribution step is performed by a special participant $D\notin \mathcal {P}$ called the *dealer*, i.e., the owner of the sensitive information, who gives pieces of information (called *shares*) to the others. The secret sharing is *perfect* if the participants of an unqualified set jointly cannot compute any information about the secret. The set of the *qualified* sets, $\mathcal {A}\subseteq 2^\mathcal {P}$ is called the *access structure*, and it is supposed to be a monotone set system, that is, if $A\in \mathcal {A}$ and $A\subseteq B$ then $B\in \mathcal {A}$ as well. The *complexity* of a secret sharing scheme is the ratio between the size of a share of maximal length and the size of the secret, where the size of the share or secret is the length of the string representing it, respectively. An access structure is *ideal* if there exists a secret sharing scheme realizing it with complexity 1, or in other words, the size of the secret and each share is the same, i.e., both are chosen from a set containing elements of the same size.

Widely used special cases of access structures are the *t*-*threshold* schemes. The qualified sets are those that have at least *t* elements of $\mathcal {P}$. Both Shamir’s and Blakley’s secret sharing schemes realize threshold access structures. The former uses polynomials to share and Lagrange interpolation to recover the secret, while the latter uses linear subspaces of a vector space. Both of these schemes are ideal. A secret sharing scheme is *linear* if the reconstruction of the secret from the shares is a linear mapping. The main goal of this paper is to propose ideal linear constructions for generalized threshold schemes. Note that, however, more general linear constructions are proposed in [3, 12]. In this paper, we consider the following:

### Example 1

Assign the vectors $d,v_i\in \mathbb {F}_q^k$ for the dealer and the participants for $i\in \mathcal {P}$, where ${\mathbb F}_q$ is the finite field of *q* elements. A (one-dimensional) *linear secret sharing generated by* $G=(d,v_1,\dots ,v_{|\mathcal {P}|})$ is the following: the dealer chooses $e\in \mathbb {F}_q^k$ uniformly at random and let the secret be the inner product of vectors *e* and *d* and the share of participant *i* be the inner product of vectors *e* and $v_i.$

The main importance of the above construction arises from the following expressive characterization result observing the connection between the perfectness of the secret sharing and the linear (in)dependence of the vectors:

### Theorem 1

(Blakley and Kabatianskii [3]) A linear secret sharing scheme generated by $G=(d,v_1,\dots ,v_{|\mathcal {P}|})$ represents an ideal perfect secret sharing scheme realizing $\mathcal {A}$ if and only if the following conditions hold:

1. $\forall X\in \mathcal {A}$ the vector *d* is a linear combination of the vectors $v_x, x\in X$;
2. $\forall Y\notin \mathcal {A}$ the vector *d* is not contained in the subspace generated by vectors $v_y, y\in Y.$

As a consequence of this result, the main goal of this paper is to find a way to assign the dealer and the participants such that the respective vectors fulfill the above requirements for a given family of particular access structures.

### Motivation

A notable family of generalizations for threshold schemes is the *multilevel schemes*. In a multilevel scheme, the participants are composed of a partition on $\mathcal {P}$ whose elements are called *levels*, and all the members within the same level are equivalent from the secret sharing point of view. One can assign different thresholds to the (combination of) levels in various ways. Within this paper, we investigate the following version motivated by the hierarchical access policies of organizations.

In *hierarchical* threshold access structures with *m* disjoint levels, let $\mathcal {P}=\bigcup _{i=1}^m\mathcal {L}_i$ and let $t_1<t_2<\dots <t_m$ be a sequence of thresholds. There are two main variants of generalized threshold schemes based on the logical relation of the conditions.

In *conjunctive* $(t_1,\dots ,t_m)$-*hierarchical schemes* the access structure is the following:

1 $$\begin{aligned} \mathcal {A}=\left\{ A\subseteq \mathcal {P}:\left| A\cap \left( \bigcup _{j=1}^i\mathcal {L}_j\right) \right| \ge t_i,\quad \mathrm {\ for \ all\ } 1\le i\le m\right\} . \end{aligned}$$

The other well-studied version, called *disjunctive* $(t_1,\dots ,t_m)$-*hierarchical schemes*, is weaker in the sense that it requires fulfilling the condition for at least one threshold only. Within this work, we are focusing on the former stricter version, i.e., on conjunctive schemes.

### Related work

Though the literature on hierarchical secret sharing is extensive, a great majority of the papers are devoted to disjunctive schemes. In the conjunctive case, some constructions are proposed for small parameters—namely 2 and 3 levels—like a (1, 3)-scheme by Fuji-Hara and Miao [4], a (1, *k*)-scheme by Shima and Doi [8], a $(1,n+1)$-scheme and 3 levels $(1,2,n+1)$-schemes by Ligeti et al. [6]. On the other hand, there are only a few general solutions based on interpolation techniques by Tassa [9], Tassa and Dyn [10], Ballico et al. [1], and on MDS codes by Tentu et al. [11].

The main contribution of this paper is to propose a novel construction based on finite geometry arguments for an arbitrary number of levels and threshold values such that the size of the underlying finite field can be significantly smaller compared to the best-known general solution of Tassa and Dyn [10]. The construction yields an ideal secret sharing scheme, which is also information theoretical perfect in the sense that there is no negligible probability of success for computing the secret even if the participants within any non-qualified subset have unlimited computational power.

## New results

Within this section, we describe our proposed geometric constructions. The ideas are non-trivial generalizations of results [6], which contain special cases of 2 and 3 levels with extreme thresholds $t_1=1$ and $t_1=1, t_2=2$, resp.

We will need the following definition.

### Definition 1

An *arc* in the *n*-dimensional projective space $\textrm{PG}(n,q)$ over ${\mathbb F}_q$ is a point set with no $(n+1)$ points in a hyperplane.

There are many results concerning arcs, see e.g. [5]. What we are going to use is the fact that the so-called *normal rational curve* is always an example of an arc. (We remark that, in most cases, the normal rational curves are of the largest possible size, or they are close to being it.) We define it here with homogeneous coordinates.

### Definition 2

A *normal rational curve* is a pointset in $\textrm{PG}(n,q)$, projectively equivalent to

$$\{(1,t,t^2,\dots ,t^n): t\in {\mathbb F}_q\} \cup \{ (0,0,\dots ,0,1) \}.$$

Note that this is a curve of size $q+1$.

Now we are ready to formulate and prove the main result.

### Theorem 2

Given integers $1\le t_1< t_2< \dots < t_m$, $n+1=t_m$, there exists a geometric $(t_1,t_2,\dots ,t_{m-1},t_m)$-scheme of *m* levels in $\textrm{PG}(n,q)$.

### Proof

We are going to construct a geometric scheme. In a suitable projective space over a finite field of order *q*, we will build a pointset corresponding to the vectors of Theorem 1. It consists of *m* levels $\mathcal {L}_1, \mathcal {L}_2, \dots , \mathcal {L}_m$. A valid subset should contain at least $t_i$ elements from $\mathcal {L}_1\cup \mathcal {L}_2\cup \dots \cup \mathcal {L}_i$ for each $i=1,2,\dots ,m$, for the given integers $1\le t_1< t_2< \dots < t_m$, where $n+1=t_m$. In $\textrm{PG}(n,q)$, we will choose our sets as follows. (We will think about the values $c_i$ (and *c*) as given constants, possibly depending on *n* and *m*; we will decide about them later, in Sect. 3.)

- Let $\textrm{PG}(n,q)=H_1\supset H_2\supset H_3\supset \dots \supset H_m$ be a chain of projective subspaces of dimension $n> n-t_1> n-t_2> \dots > n-t_{m-1}$ resp.;
- $\mathcal {L}_i$ will be a subset of size $|\mathcal {L}_i|=c_iq^{1/n}$, contained in an arc (e.g. a so-called normal rational curve) in $B_i=H_i\setminus H_{i+1}$ for $i=1,2,\dots ,m-1$;
- $\mathcal {L}_m$ will be a subset of size $|\mathcal {L}_m|=c_mq^{1/n}$, contained in an arc, e.g. a normal rational curve in $H_m$;
- furthermore, a set $\mathcal{D}\subset H_1$ of size $cq^n$ will be determined below, such that the *dealer*, i.e. a point *d* will be chosen from $\mathcal D$.

We will calculate up to an order of magnitude only. First, we choose a normal rational curve in $H_1$, then $\mathcal {L}_1$ from the points of this curve, of size $c_1q^{1/n}$.

Consider all the hyperplanes spanned by any *n* points of $\mathcal {L}_1$. There are roughly ${c_1^n\over n!}q$*n*-tuples, so if ${c_1^n\over n!}$ is smaller than 1 then these hyperplanes will not contain every $(n-t_1)$-dimensional subspace of $H_1$. (For instance, these hyperplanes cannot cover all the points of $H_1$, and through an uncovered point any $(n-t_1)$-dimensional subspace is fine.)

So take an $(n-t_1)$-dimensional subspace $H_2\subset H_1$ which is not contained in any of these hyperplanes and which, furthermore, is also disjoint from $\mathcal {L}_1$. Then remove from $H_2$ the points in the intersection with the hyperplanes spanned by any *n* points of $\mathcal {L}_1$. There are roughly ${c_1^n\over n!}q$*n*-tuples, each spanned hyperplane intersects $H_2$ in roughly $q^{n-t_1-1}$ points, hence this way we remove at most ${c_1^n\over n!}q^{n-t_1}$ points from $H_2$ (which has roughly $q^{n-t_1}$ points).

In $H_2$, a normal rational curve (which is an arc) has roughly *q* points, so an “average one” contains ${c_1^n\over n!}q$ deleted points. (One can argue as follows: let *N* be the set of normal rational curves in $H_2$ and $D_2$ the set of deleted points. The number of incident pairs (curve, point of $H_2$) is $|N|(q+1)$. So the number of curves through a point of $H_2$ is $|N|(q+1)\over |H_2|$. Hence the number of incident pairs (curve, point of $D_2$) is $|N|(q+1)|D_2|\over |H_2|$. It means that there is a curve with at most ${(q+1)|D_2|\over |H_2|}\lesssim {q\cdot {c_1^n\over n!}q^{n-t_1} \over q^{n-t_1}}$ points of $D_2$.)

So if ${c_1^n\over n!}$ is significantly less than 1, then there exists a normal rational curve in $H_2$ with at least $(1-{c_1^n\over n!})q$ non-deleted points and $\mathcal {L}_2$ can be chosen from it with cardinality $c_2q^{1/n}$.

Next, take an $(n-t_2)$-dimensional subspace $H_3\subset H_2$ which is disjoint from $\mathcal {L}_1\cup \mathcal {L}_2$ (and is not contained in the span of any *n*-tuple of them, similarly as above) and remove from $H_3$ the points in the intersection with the hyperplanes spanned by those subsets of $\mathcal {L}_1\cup \mathcal {L}_2$, which consist of *n* points such that at least $t_1$ of them are from $\mathcal {L}_1$.

This way, we remove at most ${(c_1+c_2)^n-c_2^n\over n!}q^{n-t_2}$ points, so if it is significantly less than $q^{n-t_2}$ then there exists a normal rational curve in $H_3$ with at least $(1-{(c_1+c_2)^n-c_2^n\over n!})q$ non-deleted points and $\mathcal {L}_3$ can be chosen from it with cardinality $c_3q^{1/n}$.

And so on, in general, for $i=2,\dots ,m-1$, take an $(n-t_i)$-dimensional subspace $H_{i+1}\subset H_i$ which is disjoint from $\mathcal {L}_1\cup \mathcal {L}_2\cup \dots \cup \mathcal {L}_i$, and remove from $H_{i+1}$ the points in the intersection with the hyperplanes spanned by *n* points of $\mathcal {L}_1\cup \mathcal {L}_2\cup \cdots \cup \mathcal {L}_i$, at least $t_1$ of them from $\mathcal {L}_1$, at least $t_2$ of them from $\mathcal {L}_1\cup \mathcal {L}_2$, ..., at least $t_{i-1}$ of them from $\mathcal {L}_1\cup \mathcal {L}_2\cup \dots \cup \mathcal {L}_{i-1}$.

This way, we remove at most ${(c_1+c_2+\cdots +c_i)^n-(c_2+\cdots +c_i)^n\over n!}q^{n-t_{i}}$ points, so if it is significantly less than $q^{n-t_i}$ then there exists a normal rational curve in $H_i$ with at least $(1-{(c_1+c_2+\cdots +c_i)^n-(c_2+\dots +c_i)^n\over n!})q$ non-deleted points and $\mathcal {L}_{i+1}$ can be chosen from it with cardinality $c_{i+1}q^{1/n}$.

Finally, we will choose a subset $\mathcal D$ of $H_1\setminus H_2$ in such a way that it should contain no point from the union of hyperplanes spanned by *n* points of $\mathcal {L}_1\cup \mathcal {L}_2\cup \cdots \cup \mathcal {L}_m$, at least $t_1$ of them from $\mathcal {L}_1$, at least $t_2$ of them from $\mathcal {L}_1\cup \mathcal {L}_2$, ..., at least $t_{m-1}$ of them from $\mathcal {L}_1\cup \mathcal {L}_2\cup \cdots \cup \mathcal {L}_{m-1}$.

This way, we remove at most ${(c_1+c_2+\cdots +c_m)^n-(c_2+\cdots +c_m)^n\over n!}q^{n}$ points, so if it is significantly less than $q^n$ then there remain enough points from which we can choose our set $\mathcal D$.

One may check the subsets of $S\subset \mathcal {L}_1\cup \mathcal {L}_2\cup \cdots \cup \mathcal {L}_m$, which of them are *qualified sets* for the secret sharing scheme. If $|S|\le t_m-1=n$ is too small then it is *not* qualified: and indeed, we have not allowed any point of the space to be in $\mathcal D$ which could be generated by *n* points of $\mathcal {L}_1\cup \mathcal {L}_2\cup \cdots \cup \mathcal {L}_m$.

Now for $i=1,\ldots ,m-1$, if $s_{m-i}=|S\cap (\mathcal {L}_1\cup \mathcal {L}_2\cup \dots \cup \mathcal {L}_{m-i})|\le t_{m-i}-1$ is too small then it is *not* qualified: then it must have at least $|S\cap (\mathcal {L}_{m-i+1}\cup \cdots \cup \mathcal {L}_{m} )|\ge t_m-s_{m-i}\ge t_m-t_{m-i}+1=n+2-t_{m-i}$ points on $\mathcal {L}_{m-i+1}\cup \cdots \cup \mathcal {L}_{m}$. But these points are within the projective subspace $H_{m-i+1}$ of dimension $n-t_{m-i}$, so at most $n+1-t_{m-i}$ of them can be independent. Hence the span of *S* can be generated by a subset of *S* of size *n* and no point of $\mathcal D$ is in it.

Note that the same argument shows that in any other case (i.e. when all the threshold conditions are fulfilled, so *S* is qualified) then *S* generates the entire space, so all the points of $\mathcal D$ as well.

Summarizing: choosing any point $d\in \mathcal {D}$, it will be contained in the span of any qualified subset, but not in the span of any unqualified set. $\square$

## Discussion

The construction works if $\frac{(c_1+\cdots +c_k)^n-(c_2+\cdots +c_k)^n}{n!}q$ is significantly smaller than *q* and $t_k\le |\mathcal {L}_1\cup \mathcal {L}_2\cup \cdots \cup \mathcal {L}_k|$ for $k=2,\dots ,m$. For the former it is enough to prove that $\frac{(c_1+\cdots +c_k)^n}{n!}<1$ for $k=1,\ldots ,m$. We are going to choose the constants to fulfill these conditions. For simplicity, assume that $c_1=\cdots =c_m=c$. The inequality can be written in the form:

$$\begin{aligned} \frac{(kc)^n}{n!}<1. \end{aligned}$$

Using the Stirling formula and supposing $c\le \frac{n}{me}$ we have

$$\begin{aligned} \frac{(kc)^n}{n!}\le \frac{(k\frac{n}{me})^n}{n!}\le \frac{(m\frac{n}{me})^n}{n!}\le \frac{(\frac{n}{e})^n}{\sqrt{2\pi n}(\frac{n}{e})^n} < 1, \mathrm {\ \ as\ needed.} \end{aligned}$$

The latter condition on the relation between the thresholds and the size of levels can be written in the following simple form for $c=\frac{n}{me}$:

$$\begin{aligned} n+1=t_m\le m\frac{n}{me}q^{1/n}=\frac{n}{e}q^{1/n} \end{aligned}$$

claiming that $e^{n+1}\le q$. Note that this estimate for the size of the finite field is still an exponential bound, however, it yields a significant improvement to the best known general result proven by Tassa and Dyn [10]. Their interpolation-based construction requires $q> \sum _{k=3}^{t_m+1}k^{k+2}$, which is $O(n^{n+3})$ in the case $t_m=n+1$ here, compared to our $O(e^n)$ bound.

## References

[1] Ballico E., Boato G., Fontanari C., Granelli F.: Hierarchical Secret Sharing in Ad Hoc Networks through Birkhoff Interpolation. Advances in Computer, Information, and Systems Sciences, and Engineering pp. 157–164 (2007).

[2] Blakley G.R.: Safeguarding cryptographic keys. Proc. Nat. Comput. Conf. **48**, 313–317 (1979).

[3] Blakley E.F., Kabatianskii G.A.: Linear algebra approach to secret sharing schemes. Error Control, Cryptology, and Speech Compression LNCS **829**, 33–40 (1994).

[4] Fuji-Hara R., Miao Y.: Ideal secret sharing schemes: Yet another combinatorial characterization, certain access structures, and related geometric problems (2008). https://infoshako.sk.tsukuba.ac.jp/~fujihara/ftp/sssOct.pdf

[5] Hirschfeld J.W.P.: Projective Geometries over Finite Fields, 2nd edn Oxford University Press, Oxford (1998).

[6] Ligeti P., Sziklai P., Takáts M.: Generalized threshold secret sharing and finite geometry. Des. Codes Cryptogr. **89**, 2067–2078 (2021). 10.1007/s10623-024-01496-6 PMC11832616 39975794

[7] Shamir A.: How to share a secret. Commun. ACM **22**, 612–613 (1979).

[8] Shima K., Doi H.: A hierarchical secret sharing scheme over finite fields of characteristic 2. J. Inf. Process. **25**, 875–883 (2017).

[9] Tassa T.: Hierarchical Threshold Secret Sharing. *Theory of Cryptography, TCC 2004*. *LNCS 2591* pp. 473–490 (2004).

[10] Tassa T., Dyn N.: Multipartite secret sharing by bivariate interpolation. J. Cryptol. **22**, 227–258 (2009).

[11] Tentu A.N., Paul P., Vadlamudi C.V.: Conjunctive Hierarchical Secret Sharing Scheme Based on MDS Codes, IWOCA 2013: Combinatorial Algorithms LNCS 8288. pp. 463–467 (2013).

[12] van Dijk M.: A linear construction of secret sharing schemes. Des. Codes Cryptogr. **12**, 161–201 (1997).

